# Feeding practices and nutritional status of HIV-exposed and HIV-unexposed infants in the Western Cape

**DOI:** 10.4102/sajhivmed.v17i1.398

**Published:** 2016-05-13

**Authors:** Magdel E. Rossouw, Morna Cornell, Mark F. Cotton, Monika M. Esser

**Affiliations:** 1 Department of Paediatrics and Child Health, Family Clinical Research Unit, Stellenbosch University, Tygerberg Campus, South Africa; 2 School of Public Health and Family Medicine, University of Cape Town, South Africa; 3 Department of Pathology, National Health Laboratory Service Immunology Unit (NHLS), Stellenbosch University, Tygerberg Campus, South Africa

## Abstract

**Background:**

Optimal infant- and young child–feeding practices are crucial for nutritional status, growth, development, health and, ultimately, survival. Human breast milk is optimal nutrition for all infants. Complementary food introduced at the correct age is part of optimal feeding practices. In South Africa, widespread access to antiretrovirals and a programme to prevent mother-to-child transmission of HIV have reduced HIV infection in infants and increased the number of HIV-exposed uninfected (HEU) infants. However, little is known about the feeding practices and nutritional status of HEU and HIV-unexposed (HU) infants.

**Objective:**

To assess the feeding practices and nutritional status of HIV-exposed and HIV-unexposed (HU) infants in the Western Cape.

**Design:**

Prospective substudy on feeding practices nested in a pilot study investigating the innate immune abnormalities in HEU infants compared to HU infants. The main study commenced at week 2 of life with the nutrition component added from 6 months. Information on children’s dietary intake was obtained at each visit from the caregiver, mainly the mother. Head circumference, weight and length were recorded at each visit. Data were obtained from 6-, 12- and 18-month visits. World Health Organization feeding practice indicators and nutrition indicators were utilised.

**Setting:**

Tygerberg Academic Hospital, Western Cape. Mothers were recruited from the postnatal wards.

**Subjects:**

Forty-seven mother–infant pairs, 25 HEU and 22 HU infants, participated in this nutritional substudy. Eight (17%) infants, one HU and seven HEU, were lost to follow-up over the next 12 months. The HEU children were mainly Xhosa (76%) and HU were mainly mixed race (77%).

**Results:**

The participants were from poor socio-economic backgrounds. In both groups, adherence to breastfeeding recommendations was low with suboptimal dietary diversity. We noted a high rate of sugar- and salt-containing snacks given from a young age. The HU group had poorer anthropometric and nutritional indicators not explained by nutritional factors alone. However, alcohol and tobacco use was much higher amongst the HU mothers.

**Conclusion:**

Adherence to breastfeeding recommendations was low. Ethnicity and cultural milieu may have influenced feeding choices and growth. Further research is needed to understand possible reasons for the poorer nutritional and anthropometric indicators in the HU group.

## Introduction

### Background

Observational studies show that exclusive breastfeeding in the early months of life and continued breastfeeding with timely transition to high-quality complementary foods deliver physiological and economic benefits to mothers and maximise nutrient intake, growth, development and survival of children.^1,2,3,4,5^ The World Health Organization (WHO) recommends exclusive breastfeeding for the first 6 months of life.^6,7^ Introduction of fluids other than breast milk is associated with increased morbidity and mortality during the first 6 months of life.^8,9,10^ Complementary foods should be introduced from 6 months.^11^ Continued breastfeeding up to 24 months is advised.^11^

In the absence of interventions, 5% – 20% of infants born to HIV-infected mothers acquire HIV through breastfeeding.^11^ Given the need to reduce the risk of HIV transmission, whilst minimising other risks for morbidity and mortality, current WHO guidelines state that when replacement feeding is acceptable, feasible, affordable, sustainable, and safe, HIV-infected mothers should avoid breastfeeding completely.^11^ If not feasible, HIV-infected mothers should exclusively breastfeed for the first few months and gradually stop breastfeeding, provided that conditions for replacement feeding are in place.^12^ The 2015 South African national programme to prevent mother-to-child transmission of HIV (PMTCT) supports exclusive breastfeeding for 6 months and continued up to 12 months. Maternal antiretroviral therapy (ART) should continue until the infant is fully weaned.^13^ Formula milk was provided at public health facilities solely for PMTCT until 2013.

Optimal feeding practices are crucial for the nutritional status, health and survival of infants.^14^ Anthropometric measurements are screening tools for assessing nutritional status. The three main indicators used to define undernutrition are as follows: stunting, underweight and wasting. Stunting is associated with repeated exposure to adverse economic conditions, poor sanitation and the interactive effects of poor intake and infection.^15,16,17^ Underweight indicates a history of poor health or nutritional insult, whilst wasting is associated with recent illness and failure to gain weight or a loss of weight.^18^ Knowing the levels of stunting, underweight and wasting is important in determining the overall health of the community. In contexts of high HIV prevalence such as South Africa, it is important to understand how feeding patterns impact on the health of HIV-exposed uninfected children (HEU).

The success of the PMTCT programme has decreased HIV infection in infants, conversely increasing the number of HEU infants. To date, there has been no comparison of feeding practices between HEU and HIV-unexposed (HU) children in the Western Cape. In this study, we explored feeding practices and nutritional status in HEU and HU children over 12 months. Participants had been recruited for a pilot study of innate immune abnormalities in 2009, when antenatal dual ART was provided unless combination ART was indicated in the mother for either WHO stage 3 or 4 disease or a CD4 count at below 200 cells/mL.^19^ The aim of the study was to assess the feeding practices and nutritional status of HEU and HU infants.

## Methods

HIV-infected and -uninfected mothers and their infants were recruited from the postnatal maternity wards of Tygerberg Academic Hospital, which serves patients from lower socio-economic communities in the Western Cape. The aim of the study was to compare infectious disease morbidity and vaccine responses in HEU and HU infants over 24 months.^20,21^ Fifty-five infants, 27 HEU and 28 HU, were enrolled at 2 weeks of age in the main study. The prospective substudy on feeding practices commenced in October 2009 after 6 months on study. Five (9%) mother–infant pairs, two with HEU infants and three HU, were lost to follow-up over the first 6 months of the main study. Three (5%) HEU infants became infected and were excluded. Forty-seven mother–infant pairs (85%), comprising 25 HEU infants and 22 HU infants, participated in this nutritional substudy. Eight (17%) infants, one HU and seven HEU, were lost to follow-up over the next 12 months. A trained staff nurse consented the mothers for the nutrition study. Data were obtained from 6-, 12- and 18-month visits. A staff nurse and medical professionals conducted the nutritional questionnaire (Appendix 1) at each visit. The Human Research Ethics Committee, Faculty of Medicine and Health Sciences at Stellenbosch University (N08/10/289) approved the study protocol.

Nutritional information was obtained from the caregiver, generally the mother. Weight, length and head circumference were recorded at each visit. Sociodemographic questions were included in the nutrition questionnaire. Caregivers were asked to answer yes or no to questions regarding nonnutritional foods, that is, salty snacks and sugar-containing snacks and drinks, given to the infant and the current alcohol and tobacco (smoking) use of the caregiver (the mother).

We used simple rapid-assessment techniques with the WHO indicators^22^ to assess feeding practices.

### World Health Organization indicators^22^

#### Early initiation of breastfeeding

The proportion of children who were put to the breast within 1 hour of birth.

#### Exclusive breastfeeding under 6 months

The proportion of infants 0–5 months of age who were fed exclusively with breast milk.

#### Continued breastfeeding at 1 year

The proportion of children 12–15 months of age who were fed breast milk.

#### Introduction of solid, semisolid or soft foods

The proportion of infants 6–8 months of age who received solid, semisolid or soft foods.

#### Minimum dietary diversity

The proportion of children 6–23 months of age who received foods from four or more food groups per day. The seven foods groups used for this indicator were grains, roots and tubers, legumes and nuts, dairy products (milk, yoghurt and cheese), flesh foods (meat, fish, poultry and liver or organ meats), eggs, vitamin A–rich fruits and vegetables and other fruits and vegetables.

#### Children ever breastfed

The proportion of children who were ever breastfed.

#### Bottle-feeding

The proportion of children 0–23 months of age who were fed with a bottle, regardless of whether the infant was breastfed.

#### Milk-feeding frequency for non-breastfed children

Proportion of non-breastfed children 6–23 months of age who received at least two milk feedings per day.

The WHO Anthropometric calculator application (version 3.2.2, January 2011) was used for *z*-score calculations of weight/age, length/age, weight/length and head circumference/age. Anthropometry defined^23^ as underweight: below -2 standard deviations (SD) from median weight for age of reference population; wasting: below -2 SD from median weight for height of reference population; and stunting: below -2 SD from median height for age of reference population. Feeding practices and nutritional status indicators were reported as median (med) and interquartile range.

## Results

Participants were recruited over a 16-week period from March to June 2009. Forty-seven mother–infant pairs (25 HEU and 22 HU infants) participated in this nutritional substudy (Table 1). Eight (17%) infants, one HU and seven HEU, were lost to follow-up over the next 12 months on the nutrition substudy.

**TABLE 1:** Sociodemographic characteristics of HIV-exposed uninfected and HIV-unexposed infants at enrolment in nutrition study at 6 months of age.

Characteristics	HEU (*n* = 25)			HU (*n* = 22)		
	
	*n*	%	IQR	*N*	%	IQR
Males	7	28	-	12	55	-
Ethnicity: Mixed race people	6	24	-	17	77	-
Ethnicity: White people	0	-	-	1	5	-
Ethnicity: Black people	19	76	-	4	18	-
Inhabitants per household median	6	-	3−5	4	-	4−7
Children under 13 years median	2	-	1−2.5	2	-	1−3
Number employed in household median	1	-	1	1	-	1−2
Running water within house	11	44	-	10	50	-
Ablution within house	12	48	-	10	50	-
Mothers using alcohol	1	4	-	5	23	-
Mothers smoking	7	28	-	16	73	-

HEU, HIV-exposed uninfected children; HU, HIV-unexposed children; IQR, interquartile range.

The HEU children were mainly Xhosa (76%) and HU were mainly mixed race (77%). Occupation density and number of young children in the households were similar. Nearly half of the households had running water and ablution facilities inside the house. More HU mothers smoked (73% versus 28%) and used alcohol (23% versus 4%).

In the HEU group, only one mother initiated breastfeeding and was still breastfeeding at 18 months (Table 2). In the HU group, all mothers initiated breastfeeding with over half starting within an hour of delivery. At 12 and 18 months, 62% and 52% of the HU mothers were still breastfeeding. No infant was exclusively breastfeeding at 6 months. In the HEU group, there was 100% adequate formula milk frequency at 6 months, dropping to 80% at 12 and 18 months. The HU group had a similar trend but with a later time decline (100% at 12 months and 80% at 18 months).

**TABLE 2:** Feeding practices by World Health Organization indicators 19 of HIV-exposed uninfected and HIV-unexposed children.

WHO indicator	Study visit	HEU		HU	
	
		*n*	%	*n*	%
Ever breastfed	-	1	4	22	100
Early initiation of breastfeeding	Confirmed	0	-	12	55
	Not initiated early	1	100	6	27
	Unknown	0	-	4	18
Continued breastfeeding at	6 months	1	4	13	59
	12 months	1	5	13	62
	18 months	1	6	11	52
Exclusive breastfeeding at 6 months	-	0	-	0	-
Adequate milk frequency	6 months	24	100	Not applicable	-
	12 months	16	80	6	100
	18 months	15	83	8	80
Bottle-fed	12 months	20	95	13	72
	18 months	14	93	15	79
Food introduction at 6 months	-	19	76	21	96
Minimum dietary diversity	6 months	5	20	10	46
	12 months	13	65	10	56
	18 months	11	61	14	67
Nonnutritional foods^a^	6 months	8	32	15	68
	12 months	12	52	20	100
	18 months	18	100	21	100

HEU, HIV-exposed uninfected children; HU, HIV-unexposed children.

HEU: 6 months: *n* = 25; 12 months: *n* = 23; 18 months: *n* = 18; HU: 6 months: *n* = 22; 12 months: *n* = 21; 18 months: *n* = 21.

a Nonnutritional foods (salty snacks and sugar-containing snacks and drinks).

No information was available on bottle-feeding at 6 months because of misinterpretation of the survey question. However, all but one HEU infant were assumed to bottle-feed, as only one mother was breastfeeding. Almost all HEU infants were bottle-fed from 12 to 18 months. Amongst the HU infants, 72% and 79% were bottle-fed at 12 and 18 months.

At 6 months, 96% of HU and 76% HEU infants had solid, semisolid or soft foods introduced. At 6 months, 46% of HU and 20% HEU had minimum dietary diversity. By 12 months, it was 65% for HEU and 56% for HU and by 18 months it was 61% and 67%, respectively, for HEU and HU.

Sixty-eight percent of HU and 32% of HEU infants were fed nonnutritional foods at 6 months increasing to all HU and about half the HEU infants at 12 months. By 18 months, all infants were receiving nonnutritional foods.

The HU group had poorer anthropometry (Table 3) and nutritional indicators than the HEU group at 6 and 18 months (Table 4). No HEU infants were underweight at any time point. Amongst the HU, 2 (9%) were underweight at 6 months, 3 (14%) at 12 months and 2 (10%) at 18 months. Amongst HU, 6 (27%) were stunted at 6 months, declining to 4 (19%) at 12 and 18 months. One HEU infant was wasted at 12 months and one HU infant was wasted at 6 months.

**TABLE 3:** Anthropometry of HIV-exposed uninfected and HIV-unexposed infants.

*z*-score	Months	HEU median	IQR	HU median	IQR
Weight/age	6	−0.07	−0.67 to 0.92	−0.96	−1.52 to 0.32
	12	0.04	−0.61 to 1.15	−0.39	−1.24 to −0.18
	18	0.15	−0.53 to 1.33	−0.31	−1.47 to 0.12
Length/age	6	−0.4	−1.07 to 0.09	−0.93	−2.30 to 0.07
	12	−0.09	−0.8 to 0.73	−0.39	−1.82 to 0.71
	18	−0.29	−0.72 to 0.64	−0.94	−1.64 to 0.36
Weight/length	6	1.09	−0.37 to 1.56	0.08	−0.78 to 0.87
	12	0.17	−0.46 to 1.27	−0.34	−1.04 to 0.81
	18	0.61	−0.33 to 1.21	−0.27	−1.02 to 0.62
Head circumference/age	6	0.53	0.02 to 1.07	−0.32	−1.05 to 0.15
	12	0.37	−0.02 to 1.11	−0.66	−1.22 to 0.34
	18	0.54	−0.50 to 1.15	−0.42	−0.93 to 0.51

HEU, HIV-exposed uninfected children; HU, HIV-unexposed children; IQR, interquartile range.

**TABLE 4:** Nutritional indicators of HIV-exposed uninfected and HIV-unexposed infants.

Months	Study group	*n*	Underweight		Stunted		Wasted	
		
			*n*	%	*n*	%	*n*	%
6	HEU	25	0	-	2	8	0	-
	HU	22	2	9	6	27	1	5
12	HEU	23	0	-	0	-	1	4
	HU	21	3	14	4	19	0	-
18	HEU	18	0	-	0	-	0	-
	HU	21	2	10	4	19	0	-

HEU, HIV-exposed uninfected children; HU, HIV-unexposed children.

## Discussion

In this small study, we documented detailed feeding history in HEU predominantly Xhosa infants and HU predominantly mixed race infants. In both groups, adherence to breastfeeding recommendations was low and there was suboptimal dietary diversity. However, there were significant differences between groups in the practices and outcomes.

Breastfeeding occurred in all HU and in one HEU infant(s). The mainly formula-fed HEU infants had a significant decrease in milk frequency after 6 months, coinciding with no access to free formula after this age. We noted a high rate of sugar- and salt-containing snacks given from a young age in both groups. The HU group had poorer anthropometric and poorer nutritional indicators not explained by nutritional factors alone. Alcohol and tobacco use were much higher amongst the HU mothers. All these factors (ethnicity, smoking and alcohol use) may have played a role in the difference in anthropometric and nutritional indicators between the two groups. Because of these confounding factors and small sample size, statistical analyses were not included.

The rate of breastfeeding was extremely low in HEU infants, possibly because free formula milk through public health facilities for the first 6 months of life for all HEU was standard of care at the time. All HU infants were initiated on breastfeeding, whilst the provincial average was 87.1% in 2003–2004,^24^ suggesting some success of the baby-friendly initiative.^25^ In contrast, the proportion of mothers reporting initiation of breastfeeding within an hour after birth was lower than the provincial figure (55% versus 69.3%)^24^ and the 95%^26^ found in another Western Cape study, whilst other studies reported that few infants were breastfed within 1 hour after birth.^27^ Late initiation is a concern as neonatal mortality may increase markedly with increasing delay in initiating breastfeeding.^28^

In this study, no infant was exclusively breastfed *at* 6 months. This is lower than the national average of 8.5%^24^ of infants exclusively breastfed *until just under* 6 months. It is generally accepted that the proportion of children who are exclusively breastfed *until just under* 6 months of age is lower than the number derived from the indicator of current status at 6 months,^22^ which may explain our finding. South Africa has one of the lowest rates of exclusive breastfeeding in the world.^29,30^ Reasons for this low rate are complex but include longstanding cultural practices, historical lack of promotion of breastfeeding because of high HIV prevalence and the provision of free formula milk through the PMTCT programme.^31^

Continued breastfeeding at 1 year was similar to the national proportion.^24^ Our study does not include data at 20–23 months, the WHO indicator for continued breastfeeding at 2 years. However, over 50% of HU infants were still breastfed at 18 months compared with 30.6% at 2 years nationally.^32^ In contrast, in the developing world, about 86% of infants 6–11 months were still breastfed, ranging from 92% and 88% in Africa and Asia, respectively, to 60% in Latin America and the Caribbean.^30^ For children 12–23 months of age, the prevalence of continued breastfeeding dropped to about 70% and 72% in Africa and Asia, respectively.^30^

Adequate milk frequency for HEU infants decreased significantly from 100% at 6 months to 80% at 12 and 18 months. This may have been due to the provision of free formula milk to all HEU infants for the first 6 months of life at the time of the study. Poorer caregivers may struggle to pay for formula milk thereafter^26^ or may have chosen to spend their money on other forms of nutrition. The current policy puts more emphasis on social circumstances (replacement feeding only when it is acceptable, feasible, affordable, sustainable and safe) and should help to prevent inadequate milk frequency for HEU infants in future.

The proportion of infants bottle-fed in the study exceeded national figures; 72% of HU infants at 12 months compared to 40% nationally at 12–15 months. Furthermore, 79% of this group were bottle-fed at 18 months compared to 27% at 16–19 months nationally.^32^ Information on bottle-feeding is useful because of the potential interference of bottle-feeding with optimal breastfeeding and the association between bottle-feeding and increased diarrhoeal disease morbidity and mortality.^22^

Early introduction of complementary food is common in many developing countries^33,34^ and in this study. The lower percentage in the HEU group might reflect nutrition counselling input from the clinic staff as these mothers had monthly clinic visits for formula supply in addition to immunisation visits.

Dietary diversity was inappropriate during the first few months after weaning. Dietary diversity is essential as inadequate complementary feeding at 6 months of age is associated with impaired growth and increased stunting during the next 12 months.^35^ Children aged 6–24 months are at the greatest risk from poor feeding practices.^36,37^ Minimum dietary diversity was better in the HU group compared to HEU at 6 months, poorer at 12 months, and almost equal at 18 months. The later introduction of complementary food in the HEU group might explain the poorer dietary diversity at 6 months.

All the HU were given snacks and/or drinks containing sugar and salt at 12 months and HEU infants by 18 months. The widespread use of such goods at a young age may increase risk of elevated blood pressure^38^ and obesity later in life.^39^ High-sugar foods displace whole foods (e.g. soft drinks displace milk and juice consumption) and contribute to nutritional deficiencies, adding empty kilojoules^40^ as they reduce dietary diversity. Furthermore, sugar contributes to dental caries.^41^ The Road to Health booklet already contains basic Health Promoting Messages. Health professionals at primary care should highlight this advice to mothers.

The HU group had poorer nutritional and anthropometric indicators than the HEU group, despite the higher infectious morbidity already described in the HEU group.^21^ The higher infectious morbidity by 12 months may be attributed to deficient immunity rather than their diet. In a recent study of premature infants from the same demographic area, the HEU infants also had better anthropometric indicators than the HU group.^42^ No difference in 2-year rates of adverse health outcomes between early-weaned breastfed and formula-fed children born to HIV-infected mothers has been reported.^43^ A study from Latin America found the association between stunting and feeding practices generally weaker and less consistent during the first year of life, increasing gradually with age.^44^ Stunting was more prevalent in both groups studied as observed in the national food consumption survey in 1999 where 10.3% children 1–9 years were underweight and 21.6% were stunted.^45^

A much higher proportion of the mothers in the HU group (23% versus 4%) reported alcohol use. Excessive alcohol consumption remains a serious social and public health problem in the Western Cape. The prevalence of risky drinking (more than two drinks per day for women) is higher in the Western Cape Province than in all the other provinces (9% compared with ≤ 5%), with mixed race women having higher levels than other communities (12.6% compared with ≤ 2%).^30^ Evidence regarding the negative effects of heavy drinking in pregnancy is well established. Even low levels of maternal alcohol consumption have a negative association with foetal growth.^46^

In our study, 73% of the mothers of HU infants compared to 28% of the HEU group reported smoking. The Western Cape Province has the highest prevalence of smoking of all the provinces: 44.7% of men and 27% of women including a large proportion of pregnant women.^47^ Smoking reduces weight, length and head circumference at birth^48^ and during the first 2 years of life, independent of several confounding factors.^49^ Ethnicity and the habits of the mother (smoking and alcohol use) during pregnancy and breastfeeding likely affected the difference in anthropometry and nutrition indicators between the two groups.

### Limitations

This study had a small sample size with various confounding factors in both groups. Interviewer standardisation and guidelines for use of questionnaires were not strictly controlled and some misinterpretation of the questionnaire is possible. In our opinion, even though the data are from 2009, it remains relevant to feeding practices and social issues impacting on healthy growth in the current social and economic climate.

## Conclusion

The study found disturbing information regarding feeding patterns and growth in two different cultural groups with similar economic surroundings. Infants in both groups received nonnutritional foods with high sugar and salt content, emphasising a general lack of nutritional awareness. More education and more counselling are imperative. Further research is needed to understand reasons for the poorer nutritional and anthropometric indicators in the HU group. The role of smoking and alcohol use during pregnancy and breastfeeding and other potential confounders require further investigation.
